# An Effective Synthesis Method for Tilorone Dihydrochloride with Obvious IFN-α Inducing Activity

**DOI:** 10.3390/molecules201219781

**Published:** 2015-12-02

**Authors:** Junren Zhang, Qizheng Yao, Zuliang Liu

**Affiliations:** 1 School of Chemical Engineering, Nanjing University of Science and Technology, Nanjing 210094, China; zjr1108@njau.edu.cn; 2 Laboratory of Veterinary Pharmacology and Toxicology, College of Veterinary Medicine, Nanjing Agricultural University, Nanjing 210095, China; 3 School of Pharmacy, China Pharmaceutical University, Nanjing 210009, China; qz_yao@163.com

**Keywords:** synthesis, 2,7-dihydroxyfluoren-9-one, tilorone, methyl esterification, IFN-α

## Abstract

Tilorone dihydrochloride (**1**) has great potential for inducing interferon against pathogenic infection. In this paper, we describe a convenient preparation method for 2,7-dihydroxyfluoren-9-one (**2**), which is a usual pharmaceutical intermediate for preparing tilorone dihydrochloride (**1**). In the novel method, methyl esterification of 4,4′-dihydroxy-[1,1′-biphenyl]-2-carboxylic acid (**4**) was carried out under milder conditions with higher yield and played an important role in the preparation of compound **2**. The structures of the relative intermediates and target compound were characterized by melting point, IR, MS, and ^1^H-NMR. Furthermore, the synthesized tilorone dihydrochloride exhibited an obvious effect on induction of interferon-α (IFN-α) in mice within 12 h, and the peak level was observed until 24 h. This fruitful work has resulted in tilorone dihydrochloride becoming available in large-scale and wide application in clinics, which has a good pharmaceutical development prospects.

## 1. Introduction

**Figure 1:**
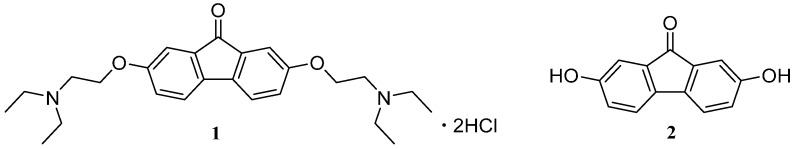
Structures of tilorone dihydrochloride (**1**) and 2,7-dihydroxyfluoren-9-one (**2**).

**Scheme 1:**
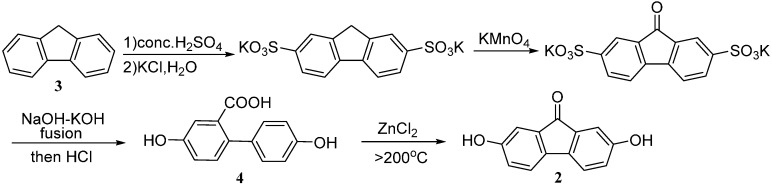
Traditional synthetic route of 2,7-dihydroxyfluoren-9-one (**2**).

Tilorone dihydrochloride (**1**, Figure 1) was first reported to have antivirus activity in mice in 1970 [1,2]. Moreover, it has been gradually proved to possess extensive pharmacological functions, such as enhanced phagocytic activity of natural killer cells [3], anti-pyretic [4], anti-fibrotic [5], anti-inflammatory [6,7,8,9,10], anti-virus [11,12], anti-tumor [13,14,15], as well as interferon induction [16,17,18]. All the effects are attributed to its planar structure of a modification of the fluorenone skeleton and amino side chain [15,19,20,21]. The skeleton comes straight from 2,7-dihydroxyfluoren-9-one (**2**, Figure 1), which can be substituted by appropriate dialkylaminoalkyl halides to give tilorone and other compounds [15]. Compound **2** has been maturely synthesized by *9H*-fluorene (**3**) in several steps as outlined in Scheme 1 [22,23,24]. However, the straight cyclization in the last step is hardly carried out by a solid-solid reaction, which will be not suitable for manufacture on a large scale. Meanwhile, a temperature higher than 200 °C is required, resulting in low purity, a great deal of energy consumption, a tendency to get out of control to burst, and the yield of pure compound **2** is not over 80%. Therefore, there is still a strong demand for developing a more effective approach to produce the intermediate for tilorone under milder conditions.

The antiviral effect of tilorone has been extensively investigated. Many studies had demonstrated the broad-spectrum antiviral activity of tilorone, which performed well with both oral administration and intravenous injection [1,25]. Additionally, some other studies had proved that the antiviral property was attributed to the induction of interferon in serum against viral infection. In addition, they also described the method of interferon assay—the plaque reduction assay measured as U/mL [16]—but it was not simple and was time-consuming. To improve the efficiency, rapid and accurate detections of interferon measured as pg/mL or ppm need to be developed and play an important role, especially the enzyme-linked immunosorbent assay (ELISA), which is most commonly used in the determination of different antigens, antibodies, cytokines, *etc.* [26,27,28].

In the present study, we successfully prepared tilorone dihydrochloride (**1**) via improving the traditional method in great yield. In contrast, we creatively employed methanol to synthesize ester so that we could obtain purer intermediate in a larger number with a low cost, which was suitable for industrial production. We also carried out an ELISA test to investigate the specific induction of IFN-α by the synthesized tilorone dihydrochloride to analyze kinetic features.

## 2. Results and Discussion

### 2.1 Synthesis of Tilorone Dihydrochloride

**Scheme 2:**
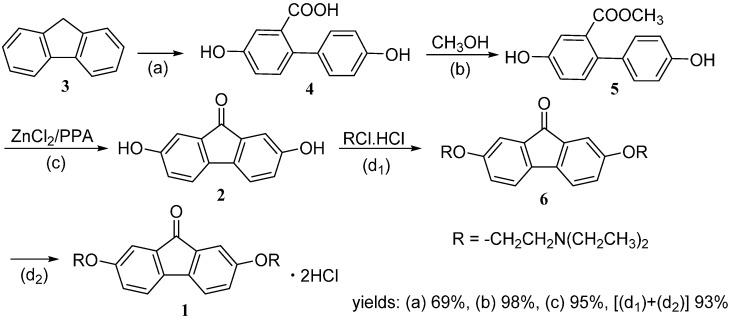
Approach to 2,7-dihydroxyfluoren-9-one (**2**) and tilorone dihydrochloride (**1**).

As shown in Scheme 2, the target compound **1** was prepared from **2**. According to the method of Andrews [22], 4,4′-dihydroxy-[1,1′-biphenyl]-2-carboxylic acid (**4**) was synthesized by the reaction of **3** in the same reaction conditions shown in Scheme 1. By examining the reaction behavior of compound **4** and ZnCl_2_ (Scheme 1), we found that compound **4** could not be fully mixed with ZnCl_2_ and converted into **2** at 200 °C, especially when the reaction scale was large. To solve the problem, we added polyphosphoric acid (PPA) in the reactants to try to make the mixture more easily, but failed. Problems still existed, including inconvenient practice and the existence of heavy material clashing due to the high temperature. In order to improve the efficiency and practicability of this reaction, we found out the following useful method. Compound **4** was firstly esterified in CH_3_OH using concentrated H_2_SO_4_ as a catalyzer under reflux for 2 h, whereby the yield of corresponding methyl ester **5** reached 98%. Then, the conversion of **5** to **2** was successfully accomplished by treatment with ZnCl_2_ and PPA at 110–120 °C in 96% yield, which reduced the temperature and improved the yield to a great degree in contrast to the direct cyclization of **4**. Finally, compound **2** was reacted with 2-diethylaminoethylchloride hydrochloride in toluene in the presence of KOH under reflux for 24 h to give **6**, which was subsequently acidified to afford **1**. In this synthetic route of compound **1** using compound **3** as a starting material, the overall yield of **1** achieved 60%.

### 2.2 Evaluation on Induction Activity of IFN-α

**Figure 2:**
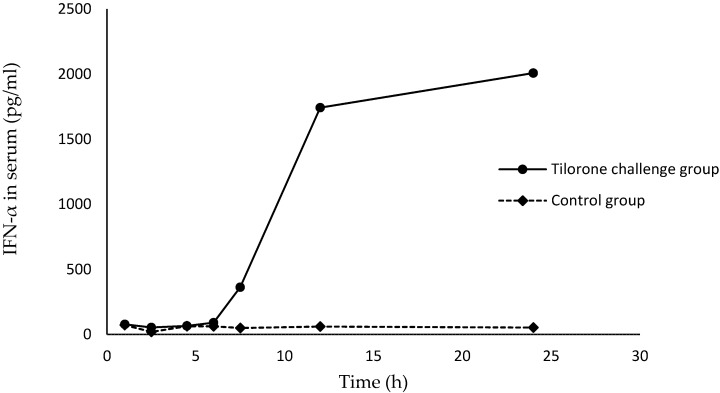
Serum IFN-α response of mice to tilorone dihydrochloride (**1**).

Tilorone dihydrochloride has shown obvious IFN-inducing and antiviral properties. In the present study, our target compound was dissolved in phosphate buffer solution (PBS) at a concentration permitting an inoculum of 250 mg/kg in specific pathogen free (SPF) mice orally. Mice were bled eight times after tilorone dihydrochloride challenge and serum was collected and diluted for determination. The data suggests that tilorone dihydrochloride stimulates a delayed but prolonged response with the probable peak levels, approximately 2000 pg/mL, being observed 12 to 24 h after gavages (Figure 2). The kinetics displayed were similar with variations of the antiviral property.

## 3. Experimental Section

### 3.1. Synthesis of Tilorone Dihydrochloride *(**1**)*

#### General

Every reaction was monitored, and the endpoint of the reaction was checked by TLC performed on GF-254 silica gel plates with visualization by UV light. Melting points were measured on an YRT-3 temperature apparatus (Tianjin Jing Tuo instrument technology, Tianjin, China) and uncorrected. IR spectra were performed on a IR spectra were recorded on a VERTEX 80 instrument (Bruker Corporation (Beijing, China). Mass spectra were determined on a VG Auto Spec-3000 spectrometer (VG instruments, Kuala Lumpur, Malaysia) and reported as *m*/*z*. The ^1^H-NMR spectral data were recorded on a Bruker Avance 400 NMR spectrometer (Bruker Corporation (Beijing), Beijing, China) and chemical shifts were reported in ppm (δ) relative to TMS as an internal standard.

*4,4′-dihydroxy-[1,1′-biphenyl]-2-carboxylic acid* (**4**). Compound **3** (200 g, 1.20 mol) was dissolved in concentrated H_2_SO_4_ (570 mL) at 110–115 °C. After 2 h of vigorous stirring, the solution was allowed to cool and water (1.7 L) was added. Then the solution was filtered. To the filtrate KCl (400 g) was added and the solution was stirred at room temperature for 30 min. Then the precipitate was filtered and dried to give a solid (396 g, 82%), which was added in batches to a stirred solution of KMnO_4_ (208 g, 1.32 mol) in water (7.1 L), maintained at 20–30 °C for 2 h, and then filtered. The filtrate was concentrated in vacuum to 4.5 L, and cooled to 5 °C. The precipitate was filtered and dried to obtain a solid (378 g, 89%), 105 g of which (0.24 mol) was added to a mixture of NaOH (210 g, 5.25 mol) and KOH (56 g, 1.00 mol). After 1 h of stirring at 270–300 °C, the melt was allowed to cool and dissolved in water (1.5 L). The solution was acidified by addition of concentrated HCl to pH 3.0, and then the precipitate was filtered and dried. Compound **4** was obtained (52 g, 95%) in an overall yield of 69%; m.p. 269–270 °C (Lit. 270–273 °C [22]); IR (KBr), υ (cm^−1^): 3145.4–2491.3 (OH), 1693.3 (C=O); ESI-MS (*m*/*z*): 229.0 [M − H]^−^, C_13_H_10_O_4_ (MW. = 230.1); ^1^H-NMR (400 MHz, DMSO-*d*_6_), δ (ppm): 12.60 (br, 1H), 9.73 (s,1H), 9.40 (s, 1H), 7.37 (d, *J* = 8.5 Hz, 1H), 7.13 (d, *J* = 8.4 Hz, 1H), 7.06 (d, *J* = 8.4 Hz, 2H), 7.03 (d, *J* = 2.5 Hz, 1H), 6.91 (dd, *J* = 8.3, 2.5 Hz, 1H), 6.74 (d, *J* = 8.4 Hz, 2H).

*Methyl 4,4′-Dihydroxy-[1,1′-biphenyl]-2-carboxylic acid* (**5**). A mixture of **4** (50 g, 0.22 mol), concentrated H_2_SO_4_ (5 mL), and CH_3_OH (400 mL) was refluxed for 2 h and then cooled to r.t., precipitated. The precipitate was filtered and dried to give **5** (52 g, 98%); m.p. 224–226 °C; IR (KBr), υ (cm^−1^): 3291.0, 3202.8 (OH) 1692.5 (C=O), 1435.5 (CH_3_); ESI-MS (*m*/*z*): 245.0 [M + H]^+^, C_14_H_12_O_4_ (MW. = 244.1); ^1^H-NMR (400 MHz, DMSO-*d*_6_), δ (ppm): 9.80 (s, 1H), 9.42 (s, 1H), 7.18 (d, *J* = 8.3 Hz, 1H), 7.02 (d, *J* = 2.0 Hz, 2H), 7.00 (d, *J* = 2.0 Hz, 1H), 6.95 (dd, *J* = 8.4, 2.6 Hz, 1H), 6.76 (d, *J* = 2.0 Hz, 1H), 6.75 (d, *J* = 2.0 Hz, 1H), 3.58 (s, 3H).

*2,7-Dihydroxy-9H-fluoren-9-one* (**2**). A mixture of **5** (50 g, 0.20 mol), ZnCl_2_ (50 g, 0.37 mol), PPA (33 mL) was stirred at 110–120 °C for 2 h and then cooled to r.t., precipitated by addition of water (500 mL). The precipitate was filtered and dried to obtain 40 g red-brown crystallized powder (**2**). Yield 95%; m.p. 336–337 °C (Lit. 338 °C [24]); IR (KBr), υ (cm^−1^): 3388.9, 3360.6 (OH), 1700.7 (C=O); ESI-MS (*m*/*z*): 211.0 [M − H]^−^, C_13_H_8_O_3_ (MW. = 212.0); ^1^H-NMR (300 MHz, DMSO-*d*_6_), δ (ppm): 9.89 (s, 2H), 7.37 (d, *J* = 4.8 Hz, 2H), 6.90 (dd, *J* = 4.9, 2.4 Hz, 2H), 6.86 (d, *J* = 2.4 Hz, 2H).

*2,7-bis[2-(Diethylamino)ethoxy]-9H-fluoren-9-one hydrochloride* (**1**). A mixture of **2** (100 g, 0.47 mol), 2-diethylaminoethylchloride hydrochloride (300 g, 1.74 mol), KOH (225 g, 4.01 mol), water (320 mL) and toluene (1.5 L) was refluxed for 24 h. The emulsion was cooled, and then organic layer was separated, washed with KOH aq. (3 mol/L, 500 mL) and concentrated up to dryness in vacuum. The residue was dissolved in anhydrous ethanol (420 g) and acidified with gasified HCl to pH 3.0. The orange product was recrystallized with ethanol, filtered, and dried to give 200 g of compound **1** in a yield of 93%; m.p. 231–233 °C (Lit. 235–236 °C [22]); IR (KBr), υ (cm^−1^): 2943.3, 2596.5 (CH), 1708.9 (C=O); ESI-MS (*m*/*z*): 411.0 [M + H]^+^, C_25_H_34_N_2_O_3_ (MW. = 410.3); ^1^H-NMR (400 MHz, D_2_O), δ (ppm): 7.12 (d, *J* = 6.5 Hz, 2H), 6.99 (dd, *J* = 6.5, 1.6 Hz, 2H), 6.89 (d, *J* = 1.6 Hz, 2H), 4.39 (t, *J* = 3.6 Hz,4H), 3.68 (t, *J* = 3.6 Hz, 4H), 3.42 (q, *J* = 5.6 Hz, 8H), 1.44 (t, *J* = 5.8 Hz, 12H).

### 3.2. Determination of Serum IFN-α Induced by Tilorone Dihydrochloride by ELISA

SPF mice were housed under conditions of constant temperature and a 12-h light cycle, with food and water provided *ad libitum* in the Animal Experimental Center, Nanjing Agricultural University. Interferon levels were recorded on a Thermo Multiskan Ascent enzyme-labeled instrument (Thermo Fisher Scientific (Beijing, China). After tilorone dihydrochloride challenge, mice were alive just like the control group, with normal activity levels, appetite, and growth. Sample serum diluent and different concentrations of the standard were added to a micro-well plate (96 tests), which was put at 37 °C for 2 h. Biotin-conjugate diluent prepared before 30 min was added after dumping the liquid out of the plate, which would be put at 37 °C for another 1 h. The plate was washed five times after dumping the liquid and Streptavidin-HRP diluent prepared beforehand was added to the plate that would be put in the thermostat again for 1 h with no light. Substrate solution was added for a 20 min reaction after washing the plate five times. Straight after that stop solution was added to the plate, which was immediately detected for OD450 values.

## 4. Conclusions

We successfully synthesized tilorone dihydrochloride (**1**) in a great overall yield of 60%. During the reactions, the creative methyl esterification process made the conversion from **5** to **2** much more easily accomplished and it was operated under much milder conditions (acquired temperature reduced from 200 °C to 110–120 °C), whereby the **2** produced was of higher purity and in greater yield so that tilorone dihydrochloride of better quality might be obtained. On the other hand, the synthesized tilorone dihydrochloride exhibited the significant property of inducing a delayed but prolonged interferon response in mice. In summary, esterification can be a good choice for preparing an intermediate of tilorone dihydrochloride, which can be an effective medicine for clinical applications.
